# Psychopathological Risk Factors Associated with Body Image, Body Dissatisfaction and Weight-Loss Dieting in School-Age Adolescents

**DOI:** 10.3390/children8020105

**Published:** 2021-02-04

**Authors:** Antonio S. Cabaco, José D. Urchaga, Raquel M. Guevara, José E. Moral-García

**Affiliations:** 1 Faculty of Psychology, Pontifical University of Salamanca, C/Compañía, 5, 37002 Salamanca, Spain; asanchezca@upsa.es; 2 Faculty of Communication, Pontifical University of Salamanca, C/Henry Collet, 90–98, 37007 Salamanca, Spain; 3 Faculty of Education, Pontifical University of Salamanca, C/Henry Collet, 52–70, 37007 Salamanca, Spain; jemoralga@upsa.es

**Keywords:** eating disorders, adolescence, body image, weight-loss diets, body satisfaction

## Abstract

Determining the comorbidity patterns leading to suffering behavioural eating disorders (BED) that are identifiable in the early stages of life, can help prevent their becoming chronic, as well as preventing the consequences deriving from the cost and effectiveness of intervention programs. The paper focuses mainly on analysing the association between behavioural/emotional risk factors and body image, body satisfaction and diet in school children, as well as confirming whether there are differences based on sex. Several questionnaires (Strengths and Difficulties Questionnaire and several items of Health Behaviour in School-age Children) including these variables were distributed and completed by the 647 adolescents (16 year olds on average) who took part in the research project. The findings confirmed a differentiated risk profile in adolescent girls in that they had greater prevalence of emotional symptoms as a general psychopathological trait, although this was offset with their prosocial behaviour. Additionally, the findings also allowed us to conclude that the factors that predict vulnerability to BEDs are sex, the presence of emotional symptoms and social and behavioural issues. At the end of this paper, we discuss some implications and consequences that should be taken into account for future work.

## 1. Introduction

The relevance of detecting early risk factors to help clarify the origin and continuation of behavioural eating disorders (BED) is key for the early diagnosis and implementation of effective intervention strategies. Furthermore, this clinical situation is more of a priority in the younger population due to the lack of empiric evidence [1]. BED alteration patterns have been associated to risk practices (weight-loss dieting) due to body image [2], cognitive and emotional dysfunctional behaviour [3], or excess of physical exercise [4], amongst others.

The Diagnostic and Statistical Manual of Mental Disorders (DSM-5) lists various types of BEDs (anorexia, bulimia, avoidant/restrictive food intake disorder, etc.) in its latest edition (APA, 2013), but there are no indications of somatic or psychopathological comorbidities, especially at the initial stages of development [5]. The practical implications are epidemiological to optimise detection, and also have clinical consequences in terms of evolution and response to treatment [6]. It is essential to determine the indicators of the emotional and behavioural symptoms that might be associated with inadequate body representations and generate dissatisfaction in adolescents with their own bodies and, as a consequence, the need to resort to restoring actions (weight-loss dieting and purging).

One of the comorbidity issues the literature highlights as a risk factor leading to suffering a BED is dissatisfaction with the body image. This is evident both in young university students and in lower school-grade adolescents [7]. All the findings agree that this association is especially prevalent in girls [8]. Additionally, the percentages of affected individuals increase in special populations, such as ballet dancers [9], a factor that turns them into targets of epidemiological and clinical surveillance. These findings become important because they point at the body discipline parameter [10] as being the way to achieve the ideal body that is socially considered as adequate. In fact, this is even more so than other variables that generate emotional instability such as mockery or psychological harassment. In fact, there is a prospective two-year duration piece of research involving over 7000 adolescents between 13 and 15 years of age that has proven the lack of a direct impact of this type of abusive behaviour on the development of subsequent eating psychopathologies [11].

The capacity for directing prevention programs must be based on the identification and classification of risk profiles that depict the differentiating key factors. Thus, sex has been found as one of the most consistent variables in literature with evidence being found in different contexts [12,13,14,15,16,17,18]. The common factor in the conclusions of these papers is that female adolescents have a lower degree of satisfaction with their body image than that of male adolescents, with girls having an incorrect perception of their body weight (they see themselves fatter), showing greater obsession for slimness and having aspirations for BMI that are opposite to those of boys (higher in boys and lower in girls), all of which derives in a physical self-perception that is more devaluated and less attractive in girls.

The behavioural pattern that is generally followed in order to offset their perceived imbalance is mainly the adherence to weight-loss diets, either controlled or risky [19,20], with negative physical health consequences [21,22] and mental consequences [23]. It is important to point out that this sex-differentiated behavioural pattern as a reaction to body image has derived in the development of specific instruments for men [24]. The modulating role of sociocultural pressure in both sexes is very important, as it mediates in physical self-perception [25]. Age development could be added to the sex variable, involving an element of additional vulnerability due to the intrinsic changes that are present in adolescence [26,27]. The association between body image and weight-loss diets is also noticeable in special samples such as ballet dancers, where there is evidence in both sexes of a food intake pattern that is not adequate for the fulfilment of the nutritional needs the activity actually demands [28]. This aspect is related to the social representations and the beliefs around functional food that are sustained by the general population. Such beliefs do not correspond with healthy scientific evidence but with market interests that are highly influenced by advertising, and generate risky behavioural trends [29].

Given the evidence shown above, it is necessary to intervene in the modification of eating behavioural patterns and practices with the help of psycho-educational programs that have demonstrated effectiveness both in average [30] and clinical populations [31]. However, it is also important to deal with cognitive and emotional variables associated with the processing of information; promoting [3] the idea that they are at the root of unsatisfactory representations of body image as well as behavioural and social difficulties. The trends in inhibitory processing (high P3 averages in Go/NoGo tasks) with high-calorie stimuli obtained in the previous research, are similar to those found with the emotional Stroop test. This technique reveals longer response times (RT) to emotionally activating content stimuli relating to the disorder (fat, cake, vomiting, etc.), than to neutral information [32].

In the nonexperimental line of this piece of research we must highlight that in order to assess the rates of prevalence of emotional and behavioural problems in children and adolescents, various strategies have been used such as self-reporting and interviews. In other clinical research work, the interviews are the most commonly used instrument, as they allow for the performance of a formal diagnosis, whereas, in epidemiological research studies, the most habitual instrument is self-reporting. The reasons for the latter being more recommendable appear to be the amount of time required and the possibility of assessing large samples of population, although it may not contribute to the making of a formal diagnosis of the disorder. In field studies within adolescent populations, one of the instruments that is highly referred to in the literature is the Strengths and Difficulties Questionnaire (SDQ). This was developed by Goodman [33] and verified in epidemiological research studies with large samples, which allowed the prevalence indicators for mental health disorders to be established in the adolescent population in Great Britain [34], and also in children [35].

Eating disorders are amongst the most complex health problems currently being identified by healthcare professionals. This is the reason why the prevention and treatment of these pathologies at their initial stages is very important [36]. There is an association between the various ways in which eating disorders reveal themselves and certain psychological traits of the personality. Thus, rigid perfectionism is more prominent in individuals who impose eating restrictions on themselves, and impulsivity and anxiety are prominent in binge eating [37]. Emotional instability has been pointed out among the specific risk factors following the findings from longitudinal studies, which have shown that early difficulties in regulating emotions during childhood were predictive of clinical alterations relating to ED during adolescence [38]. In addition, the early habit of dieting (control of body weight due to dissatisfaction with BMI) is a behavioural predictor associated with the presence of eating disorders at later stages [39]. Additionally, all the studies mentioned previously agree that female adolescents are the more vulnerable group. It is for this reason that all interventions that include the aforementioned parameters in clinical samples (anorexia nervosa) have obtained satisfactory results by contributing to the improvement of self-perception (as it helps reduce body dissatisfaction), thus having a direct impact on the reasons for dieting [40] above mentioned.

According to the aforementioned research studies, the purpose is to prove the association between psychopathology in adolescents and the risk of early indicators of BED. As explained, the greater vulnerability to EDs in people with cognitive biases or emotional hypervigilance to food-related stimuli is relevant to understanding the early etiological mechanism. The clinical evidence with patients suffering from anorexia nervosa proves that long-term starvation behaviours are based on the fact that individuals following this behaviour develop a more inhibited response than healthy adolescents when faced with caloric stimuli [41]. On comparing it to obesity, the latter disorder shows that cognitive difficulties are more associated with difficulties in decision-making or a change of mindset [42]. In other words, the cognitive pattern in obesity is holistic or global, whilst in anorexia, it is analytical, however, emotional dysregulation plays a relevant role in treatment success [43] for both disorders. There is extensive literature on the early comorbidities of eating disorders, anxiety and depressive behaviours, even in subclinical populations [44]. The literature shows an interrelation between the cognitive variables of information processing (representation of the body image) and those of an emotional type, which, in the event of being dysfunctional (emotional lability) generate restrictive behaviours to modulate BMI [45]. Thus, we are attempting to confirm the hypothesis that the percentage of adolescents who will report emotional and behavioural symptoms at subclinical level will be high and that, within these, there will be indicators of vulnerability to the BEDs under assessment (body image and dissatisfaction and weight-loss dieting). In the same way, we believe that hypothetically, the behavioural and affective symptoms and the early risk of developing a BED will vary subject to sex in adolescents, with it being higher in the female population.

## 2. Materials and Methods

### 2.1. Participants

The sample consisted of 647 pupils in their 4th year of Obligatory Secondary Education, with greater presence of girls (55.7%) and an average of 16.1 years old (SD: 0.74). The main characteristics of the sample are summarised in Table 1.

With regards to design, it is important to highlight that this is a cross-sectional descriptive study. The sample selection was performed by a multiple-stage random sampling technique, stratified by conglomerates in Salamanca (Spain), taking as relevant variables the type of school centre (state/private) with all eight districts of the city being represented. A total of 16 school centres of the city took part in this study.

### 2.2. Instruments

A sociodemographic questionnaire and various validated specific scales were used in order to study the various dimensions. The following instruments were used:

#### 2.2.1. A Sociodemographic Questionnaire

In order to determine the sex (male and female) and age in years.

#### 2.2.2. Behavioural and Emotional Symptoms

A Strengths and Difficulties Questionnaire (SDQ) [33] was used in order to assess these dimensions of the mental health of adolescents. This instrument is a self-reporting measure that helps assess the selected dimensions, taking the latest 6 months as reference criteria. The SDQ is made of a total of 25 statements, distributed along five subscales (with five items each): emotional symptoms, behavioural issues, hyperactivity, socialisation issues, and prosocial behaviour. For all subscales, except for prosocial behaviour, the higher the score, the more the presence of emotional and/or behavioural symptoms. The first four subscales were part of a total score for Difficulties. For the purposes of this study, a Likert answer format was used with three answer options (1 “It is not true”; 3 “It definitely is true”), and therefore the score for each subscale could range between 5 and 15 points. It is important to point out that previous studies have used the 5-point *Likert* version of the SDQ, for reasons relating to score reliability, obtaining validity evidence and user satisfaction [46], showing good internal consistency (Cronbach’s α: 0.73) [33]. Nevertheless, for the purposes of this paper we decided to keep the format as per the original version established by Goodman (2001) for cross-sectional studies, or the version also used in his later publications for longitudinal studies [47]. Our decision was also supported by the fact that this it is the format used in the Health Behaviour in School Children (HBSC) study.

This instrument presents several advantages such as ease of application, briefness and a combination of negative (hyperactivity, behavioural symptoms, etc.) and positive dimensions (prosocial behaviour). Some studies validate this assessment in Spanish and yield good psychometric properties [48]. This paper determines the rates of prevalence of emotional and behavioural symptoms in a nonclinical sample (*n* = 1319) with Spanish adolescents, where we separate the differential weight that sex and age have. The findings lead us to the conclusion that a high percentage of the individuals under assessment show emotional and behavioural symptoms (between 12% and 35% for emotional symptoms), and the rate goes up to 47% for hyperactive behaviour. It is important to highlight that we are referring to subclinical levels of symptoms, given that a formal diagnosis cannot be obtained through this technique. The findings that are explained in this paper do not reveal exactly the same rates as those for other European countries, such as Germany [49], or Holland [50] or Denmark [35], but they are relevant for their use of SDQ as a means to predict psychopathological disorders, even when the differences in the SDQ scores do not necessarily imply modifications in the prevalence of the child and adolescent mental disorder in such countries [51]. What is relevant is that since the emergence of the initial research studies [52], this instrument has proven to be a useful epidemiological surveillance tool, both for child populations and adolescent populations in general, and for specific samples of children in fostering situations [53] or children with intellectual disabilities [54]. Additionally, although the SDQ has been used even to determine a dysregulating profile relating to clinical adolescents with suicidal thoughts and behaviour [55], we have not yet found any attempts to obtain a similar systematic approach in relation to the BEDs in adolescents in the literature.

#### 2.2.3. Questionnaire on Weight-Loss Diet Adherence

An item from the HBSC report, which is a study sponsored by the World Health Organisation (WHO), was used. The question was formulated as follows: “At present, are you following any kind of weight-loss diet or doing anything to lose weight?”, for which four answer options were provided: (1) No, I think my weight is adequate; (2) No, but I should lose some weight; (3) No, because I need to put on weight; (4) Yes.

#### 2.2.4. Questionnaire on Body Image and Satisfaction

In order to assess these variables, two indicators from the HBSC report were used. The first one was formulated in the following way: “Do you think your body is…? (1) Too thin; (2) A bit thin; (3) The adequate size; (4) A bit fat; (5) Too fat. This would mean that the extreme scores (near 1 or 5) would be linked to dissatisfaction with one’s own body, although they could be interpreted in different ways subject to the type of BED. The second item from the HBSC report, relating to feelings about one’s own body is an adaptation of the Body Investment Scale (BIS) by Orbach and Mikulincer (1998) BIS-1 [56], supported by later papers obtaining good levels of internal consistency, the subscale presenting a Cronbach’s alpha of 0.88 [14]. Out of the four factors making up the BIS, this study only used the first one (feelings and attitudes about one’s body image), as it is the most relevant for the purposes of the study. The subscale assesses the feelings and attitudes with regards to body image through six items, out of which three are negative or unfavourable (1 “I feel frustrated with my physical appearance”; 3 “I hate my body”; 5 “I dislike my body”) and the other three are the opposite (2 “I am happy with my appearance”; 4 “I feel comfortable with my body”; 6 “I like my appearance, in spite of my defects”). The answer options correspond to a Likert-type scale with five answer options ranging from 1 (Totally disagree) to 5 (Totally agree). In order to analyse the data, we followed the same criteria as other HBSC studies [2], classifying the individuals into three groups: low, average and high satisfaction with the same intervals (1–2.33; 2.34–3.66; 3.67–5).

### 2.3. Procedure

This study is part of a more comprehensive research project on early detection of lifestyles relating to health in adolescents under the sponsorship of the parameters used by the World Health Organisation (WHO), and was presented to headmasters, parents and participants as a piece of research on health habits. Prior to the stage of data collection, the school centres were contacted in order to obtain the relevant approval from the institution’s management, as well as the legal consent of parents and custodians. The terms and conditions for participation were detailed as follows: voluntariness, anonymity, confidentiality of the results and lack of remuneration for participating.

The HBSC questionnaire was distributed collectively to groups of students during school time and in their usual classrooms. Performance of the assessment was at all times under the supervision of the head researcher, with the help of field-trained research assistants. Additionally, the study strictly met the ethical criteria pointed out in the Declaration of Helsinki (2013 version) for research studies of this type. The study protocol was approved by the Ethics Committee for Research with Human Subjects of the Pontifical University of Salamanca on 13 November 2020.

Lastly, as inclusion criteria, in addition to acceptance of the established terms and conditions (voluntary participation, provision of legal authorisation and waiver of remuneration), the individuals were required to explicitly inform as to whether they were following any weight-loss diet or nutritional plan at the time they took the assessment or in the 6 months prior to the assessment. Failure to comply with any of the previous criteria or the incorrect or incomplete completion of the assessment were reasons for exclusion.

### 2.4. Data Analysis

Data analysis was performed through the 24.0 version SPSS statistics package. For the purpose of the statistical study descriptive statistics were used. Additionally, for hypothesis contrast, Student’s *t*-test, the effect size (D Cohen), Pearson correlations, and regression analysis (taking emotional and behavioural symptoms as continuous independent variables; body satisfaction and diet as dependent variables; finally, sex as a covariable) were performed (multiple linear regression analysis for FBI, and logistic regression for Diet and for WI). The Durbin–Watson test was calculated for the study of autocorrelation, based on the criterion that values between 1.5 and 2.5 verify the independence of the errors.

## 3. Findings

The descriptive findings from the SDQ in the five dimensions assessed by the questionnaire are reflected in Table 2. Girls reveal significantly more emotional symptoms (*p* < 0.001), and fewer behavioural (*p* < 0.001), socialisation and hyperactivity (*p* < 0.05) issues. On the other hand, girls (*M* = 13.16) more often displayed a prosocial behaviour than boys (*M* = 12.49) (*p* < 0.001).

In terms of the magnitude of the prevalence of the emotional and behavioural symptoms, as Table 3 shows, the distribution is uneven among categories. Thus, 45.8% of boys and 45% of girls show at least one difficulty in the areas under assessment; only one difficulty is shown by 34.3% of boys and 31.9% of girls, whereas there are two or more difficulties in 11.5% of boys and 13.1% of girls. On average, there are no significant differences in the number of difficulties according to sex (*p* = 0.891).

In the second assessment, i.e., the self-reported question on the adherence to diets, the findings reveal that 9.1% of boys and 16.4% of girls follow some kind of diet (Table 4). Amongst those who do not follow a diet, the boys state—to a greater extent than girls—the reason as being they believe their weight to be adequate (boys: 63.3%; girls: 47.8%). There are more girls than boys who do not follow a diet, but who think they should lose some weight (girls: 30.8%; boys: 18.9%), and there are more boys who believe they need to put on weight (boys: 8.7%; girls: 5%). There is a significant association between sex and following a diet (*p* < 0.001).

In terms of body image, only 3.4% of boys and 5.5% of girls think their weight is very far from adequate (too fat or too thin). The majority of adolescents (56.6% of boys and 46.7% of girls) think their weight is adequate. Generally speaking, girls tend to think they are overweight (a little fat or too fat: 43% of girls, 23% of boys). On the other hand, there are more girls than boys who think their body is too thin (girls: 1.9%; boys: 1%). There is a significant association between sex and body image (*p* < 0.001) (Table 5), in a way that girls appear to be more prominent at both ends of weight perception (overweight or underweight in body image).

With regards to body satisfaction (Table 6), boys reveal a significantly (*p* < 0.001) higher level of satisfaction with their bodies (*M* = 4.17) than girls (*M* = 3.64). Therefore, for the three category levels, 76.2% of boys report high satisfaction against 50.8% of girls. At the other end, 11.7% of girls report low levels of satisfaction against boys, who only show dissatisfaction in 1.7%. Additionally, in both groups there is a differentiated pattern of percentages at the medium level: 22.0% in adolescent girls and 37.5% in adolescent boys.

With regards to the prediction of an association between emotional and behavioural symptoms in adolescents and the indicators of vulnerability to BEDs (diets and body image), the correlation and multiple regression analysis (Table 7 and Table 8) show that there are several factors that enable the prediction of risk indicators. For the purposes of analysis, behavioural and emotional factors, as well as sex, were used as factors for the prediction of issues. The findings reveal that following a weight-loss diet is significantly associated with greater presence of social and behavioural issues. In addition, there is evidence of greater vulnerability in girls, since they tend to follow diets more frequently than boys. Taking the set of all predictive factors into account, following a diet can be significantly predicted (*R* = 0.291).

The negative feelings and attitudes towards body image are significantly associated with a greater presence of social and behavioural symptoms as well as with emotional symptoms. Sex is an important factor too, since girls report more negative feelings and attitudes towards their body image than boys. Taking into account the set of all factors under analysis we can significantly predict negative feelings and attitudes towards body image (*R* = 0.490).

Body image in terms of whether the weight is adequate or not is significantly associated with a greater presence of social issues, as well as with emotional symptoms. Girls—in comparison to boys—think their weight is not adequate. Taking into account the full set of factors described above, we can positively tell whether their weight is perceived as adequate or not (*R* = 0.235).

Therefore, the factors that are more significant for the prediction of vulnerability towards BEDs are sex, the presence of emotional symptoms, social issues and behavioural symptoms (the last one not being significantly associated with weight perception). Hyperactivity and prosocial behaviour are not significant factors, and therefore their relevance in the origination and continuance of the BEDs must be disregarded in terms of risk prioritisation.

## 4. Discussion

As other research studies indicate [48,50], the findings confirm that the percentage of adolescents with emotional and behavioural symptoms is high, since nearly half the sample reports to have them (around 45% with at least one difficulty in the areas under assessment). Such studies do contribute information on the quantitative sex differences in adolescents, which is a key factor we cannot confirm. Furthermore, the qualitative pattern is a different one, since there is a greater presence of emotional symptoms in adolescent girls, although, at the same time, they have more prosocial resources to overcome them. These results have been confirmed by multiple previous research studies that establish a stable pattern at this stage of life in various contexts and countries [34,35,52,53].

In terms of the specific issues relating to BEDs, there is a behavioural pattern in the practice of weight-loss diets which is nearly twice as frequent in girls. These results coincide with other studies carried out on the same line of research [12,18,57]. For the purposes of designing help programs, it is important to take into account the reasons why there are differentiated behavioural patterns in boys and girls, since girls tend to think their weight is adequate to a lesser extent and the distorted perception of their weight is rarely associated with the need to put on weight, but weight loss; boys, on the other hand, feel the opposite way. The reasons mentioned above relating to cultural pressures and the social idea of what constitutes feminine may be reasons that justify such differences [7,8,9].

The body image is in close association with this parameter, as once again there is a difference between the sexes. Girls tend to be dissatisfied, although in general the data are not alarming in terms of frequency. Therefore, we can confirm the accumulated research evidence reveals the significant association between sex and body image, which contributes an important element to be taken into account in BEDs prevention programs [2,16,19]. Likewise, the findings for the body satisfaction variable point in the same direction, since it is significantly higher in boys. Given that these two variables can be considered as mirroring and supplementary, evidence towards considering both together is justified by these and other findings in agreement with these [21,26].

In addition to the sex differentiation profile, the purpose of this paper is to take a step towards the discrimination of the indicators of vulnerability to BEDs for the purposes of a psychopathology. As a recent study demonstrates [1], following weight-loss diets is significantly associated with a greater presence of social and behavioural issues, as well as emotional symptoms. From an analytic epidemiological point of view, it is very relevant to point out that negative feelings and attitudes towards body image are significantly associated with a greater presence of social and behavioural issues, as well as emotional symptoms. In the three parameters that have been pointed out, the vulnerability of the female population is clearly a priority target because their indicators are more prevalent [13,15].

Following the indications of international organisations (UNICEF), on the basis of the legal rights that protect children and adolescents, it is essential to establish adequate eating patterns from childhood in order to prevent disease [58]. The lack of such preventative actions generates inadequate habits (at stages even previous to adolescence), with primary school children consuming food with insufficient nutritional content [59]. Besides, the assessment and intervention at a global scale must be contemplated since the variables associated with self-perception and the practice of weight-loss diets is not independent from the patterns of physical activity and the resulting BMI [60]. Nevertheless, the results of the interventions are actually generating optimism, with many medical cases of unspecified eating disorders [61] revealing improvement in the emotional situation, attitude towards food and physical activity in female adolescents taking part in mental training studies. Additionally, in patients with anorexia nervosa the change in the physical activity program has an effect on the most concerning key variable, which is the gaining of weight. Therefore, it is relevant for the preventative or clinical interventions to focus on the aforementioned key aspects (psychopathological, body image, following of weight-loss diets), as these are early factors for the origination of the BEDs.

This piece of work presents limitations that need to be pointed out in order to contribute to improved future studies. On the one hand, the age variable needs to be taken into account as a factor of development, as adolescence is a time of development towards maturity and of change in various aspects of the biopsychosocial level. On the other hand, the inherent issues of the self-reporting technique (understanding of items, rate of false positives, etc.) and the version of the assessment being used (the 3-point Likert-type original version was used, as opposed to the 5-point version used in other studies), which proves to have some restrictions in terms of the comparison of results. Finally, we think it would be advisable for subsequent research studies to analyse the body mass index of adolescents in order to see how this indicator of body composition interacts with the variables under study.

The work carried out can be expanded using specific evaluations to measure the variables associated with BEDs, such as tests of satisfaction with body image or dietary inventories. Thus, the information on these behaviours will be more dimensional and useful for intervention programs. Besides, it would be convenient to compare the answers provided by adolescents against those provided by external informants (parents or teachers) in order to enable confirmation of the potential trends with more certainty. Lastly, we believe it is necessary to combine cross-sectional studies such as this one, with longitudinal studies in order to ascertain the impact on the evolution of the prevalence rates. This way, the parameters of evolution of the risk can be determined or, to the contrary, the vulnerability of certain variables can be minimised on account of the specified evolution aspects. The findings confirm there is a relationship between variables “dieting” and “body dissatisfaction”, a relationship mediated by the presence of emotional instability with a higher risk for female adolescents. The findings from clinical samples are confirmed in the normal population, using different instruments but measuring the same variables. In view of the controversial nature of the subject, it also relies on the lesser relevance of hyperactivity or prosocial behaviour on vulnerability to restrictive eating disorders.

## 5. Conclusions

Our findings allow us to focus on our original hypothesis in terms of the importance of determining the early association of the origins, reasons and continuation of the BEDs, although we cannot establish a cause–effect association between the variables under analysis. Nevertheless, the factors herein presented as most significant for the prediction of indicators of vulnerability to BEDs are sex, presence of emotional symptoms, social and behavioural problems, with the latter not being related to body image. Other negative (hyperactivity) or positive (prosocial behaviour) variables are not significant.

Additionally, the findings regarding the association between the emotional–behavioural symptoms with risk factors, such as following a weight-loss diet or having a dissatisfactory body image will allow for the design of more effective and efficient intervention strategies and focused preventative programs on the target samples (women with emotional disorder symptoms).

## Figures and Tables

**Table 1:** Characteristics of the participants.

Characteristics of the Students		(Number) %
Sex	Boys	(286) 44.3%
	Girls	(361) 55.7%
Age	15	(348) 53.8%
	16	(208) 32.1%
	17	(789) 12.1%
	18	(13) 2%
Type of school centre	State	(389) 60.6%
	Private	(253) 39.4%

**Table 2:** Descriptors of SDQ scales according to sex.

SDQ		Sex	Symptoms (%) ^a^		*M (SD*)	Levene’s Assessment (*p*)	*t* (*p*)	D Cohen
			No	Yes				
Difficulties	Emotional symptoms	Boy	88.5	11.5	7.59 (2.08)	0.136	<0.001	0.80
		Girl	77.8	22.2	8.64 (2.23)			
	Behavioural issues	Boy	91.6	8.4	7.80 (1.86)	0.007	<0.001	0.38
		Girl	96.1	3.9	7.12 (1.70)			
	Social issues	Boy	93.4	6.6	7.03 (1.89)	0.057	0.045	0.05
		Girl	96.9	3.1	6.75 (1.62)			
	Hyperactivity	Boy	65.7	34.3	9.59 (2.20)	0.304	0.035	0.17
		Girl	69.2	30.8	9.21 (2.29)			
	Prosocial behaviour	Boy	-	-	12.49 (1.81)	0.002	<0.001	0.34
		Girl	-	-	13.16 (1.57)			

^a^ No: ≤ 10 score; Yes: > 10 score.

**Table 3:** Number of difficulties according to sex.

Difficulties	Sex		Levene’s Test (*p*)	*t* (*p*)	D Cohen
	Boy	Girl			
0	54.2	55.0			
1	34.3	31.9			
2	8.0	11.4			
3	3.5	1.4			
4	0	0.3			
*M* (*SD*)	0.608 (0.78)	0.600 (0.77)	0.986	0.891	0.01

**Table 4:** Questionnaire descriptors on diet following.

Sex	Diet				χ^2 a^	*p*	*C* ^b^
		No		Yes			
	I think my weight is adequate	But I should lose some weight	I need to put on weight				
Boy	63.3%	18.9%	8.7%	9.1%	25.7	<0.001	0.196
Girl	47.8%	30.8%	5.0%	16.4%			

^a^ χ^2^: Chi-Squared; ^b^ *C*: Contingency coefficient.

**Table 5:** Body image according to sex.

Sex	Body Image					χ^2 a^	*p*	*C* ^b^
	Yes, it is Adequate…			No, too…				
	A Little Thin	Adequate	A Little Fat	Thin	Fat			
Boy	19.2%	56.6%	20.6%	1.0%	2.4%	37.1	<0.001	0.223
Girl	8.3%	46.7%	39.4%	1.9%	3.6%			

^a^ χ^2^: Chi-Squared; ^b^ *C*: Contingency coefficient.

**Table 6:** Body satisfaction according to sex (BIS).

Sex	Body Satisfaction ^a^			*M (SD*)	Levene’s Test (*p)*	t	D Cohen
	Low	Medium	High				
Boy	1.7%	22.0%	76.2%	4.17 (0.73)	<0.001	<0.001	0.62
Girl	11.7%	37.5%	50.8%	3.64 (0.96)			

^a^ Low: 1–2.33; Medium: 2.34–3.66; High: 3.67–5.

**Table 7:** Associations between behavioural and affective symptoms (SDQ) and indicators of vulnerability to BEDs.

SDQ	Diet ^a^	Body Image: Feelings and Attitudes (BIS)	Body Image: Weight/Size ^b^
Emotional symptoms	0.084 *	−0.436 ***	0.217 ***
Behavioural issues	0.134 **	−0.115 **	0.017
Social issues	0.126 **	−0.198 ***	0.141 **
Hyperactivity	0.061	−0.054	−0.005
Prosocial behaviour	−0.027	0.020	0.021

^a^ Diet: No = 0; Yes = 1. ^b^ Body image: A bit thin/fat or Adequate = 0; Too fat/too thin = 1. * p < 0.05; ** *p* < 0.01; *** *p* < 0.001.

**Table 8:** Regression analysis: following a weight-loss diet (DIET), weight image (WI), feelings and attitudes towards body image (FABI) (dependent variables), behavioural and emotional symptoms (independent variables), adjusted to sex covariable. Multiple linear regression analysis (FBI) and logistic regression (DIET and WI).

		Unstandardised Coefficients			Standardised Coefficients				One-Way ANOVA		
		*B* ^a^	*SE* ^b^		*B* ^a^	*t* ^c^		*p* ^d^	F ^e^	*p* ^d^	
FABI ^g^	Cons.	6.202	0.231			26.84		<0.001	40.5	<0.001	
	ES ^c^	−0.138	0.016		−0.340	−8.64		<0.001			
	BI ^c^	−0.027	0.020		−0.054	−1.35		0.177			
	SI ^c^	−0.048	0.020		−0.094	−2.45		0.015			
	H ^c^	−0.002	0.015		−0.005	−0.138		0.890			
	Sex ^d^	−0.416	0.067		−0.229	−6.18		<0.001			
		*R*: 0.490; R^2^: 0.240; Adjusted R^2^: 0.235; Durbin–Watson: 1.926									
		***B*** **^a^**	***SE*** **^b^**	**Wald ^f^**	***p***		**Exp (*B*) ^g^**				
DIET ^h^	Cons.	−6.509	0.968	45.21	<0.001		0.001				
	ES ^c^	−0.027	0.059	0.209	0.648		0.973				
	BI ^c^	0.182	0.071	6.64	0.010		1.200				
	SI ^c^	0.187	0.070	7.17	0.007		1.205				
	H ^c^	0.060	0.060	1.02	0.313		1.062				
	Sex ^d^	0.958	0.276	12.05	0.001		2.607				
		Nagelkerke R Square: 0.085 (*R*: 0.291)									
WI ^h^	Cons.	−7.354	1.629	20.39	<0.001		0.001				
	ES ^c^	0.408	0.096	18.09	<0.001		1.504				
	BI ^c^	−0.120	0.120	0.99	0.321		0.887				
	SI ^c^	0.203	0.108	3.54	0.049		1.225				
	H ^c^	−0.019	0.098	0.04	0.843		0.981				
	Sex ^d^	0.101	0.446	0.05	0.821		1.106				
		Nagelkerke R Square: 0.160 (*R*: 0.40)									

Cons.: constant; ES: emotional symptoms; BI: behavioural issues; SI: social issues; H: hyperactivity; Sex (0 = boys; 1 = girls); DIET (0 = no; 1 = yes); WI (0 = adequate, a little thin/fat; 1 = too fat/thin); FABI: feelings and attitudes towards body image (continuous variable). ^a^ Regression coefficient; ^b^ Standard error; ^c^ t-test; ^d^ Significance; ^e^ F-Distribution; ^f^ Wald’s test; ^g^ exponentiation of the B coefficient ^g^ Continuous variable; ^h^ Categorical variable.

## Data Availability

All data are presented within the article.
